# Using positive deviance (PD) to reduce antibiotic resistant organisms: the Canadian PD project

**DOI:** 10.1186/1753-6561-5-S6-O50

**Published:** 2011-06-29

**Authors:** P Reason, L Rykert, TCPP Group, M Gardam

**Affiliations:** 1 Infectious Disease Prevention and Control, Ontario Agency for Health Protection and Promotion, Canada; 2 Infection Prevention and Control, University Health Network, Toronto, ON, Canada

## Introduction / objectives

5 acute care hospitals were recruited to implement PD (at 6 sites) to determine whether it can reduce healthcare-associated (HA) AROs, specifically methicillin-resistant *S. aureus* (MRSA), vancomycin-resistant Enterococci (VRE), and *C. difficile*.

## Methods

Four-month HA-ARO rates, the volume of alcohol hand rub and soap used, and the number of gowns and gloves used, were collected at baseline and then for 12 months prospectively. Social network mapping was conducted at the project start and end. Qualitative staff interviews were conducted at the project end. The percent change from baseline in quarterly HA-ARO rates were measured from September 2009 to December 2010. Process measures were collected and measured in a similar fashion.

## Results

Of the 6 sites, 5 implemented PD as planned, while one was unable to, largely due to organizational restructuring. Three of the 5 sites sustained decreases in HA-AROs of 25%, 41.2% and 63.9%. Rates at the 4^th^ site were unchanged, while the fifth site had a VRE outbreak, which resulted in a large increase in the overall HA-ARO rate. HA-MRSA decreased by 100% at 2 hospital sites; HA-VRE decreased by 100% at 2 sites; and HA-*C. difficile* decreased at 3 sites by 53%, 51.9% and 23%. The 1 site that measured hand hygiene compliance had a 53.2% rate increase. Interestingly, decreasing HA-ARO rates did not clearly correlate with the process indicators.

## Conclusion

PD has been successfully used in a number of settings facing complex problems. We have shown it to be successful in reducing HA-AROs in Canadian acute care facilities where the organizational climate allowed it to be implemented.

## Disclosure of interest

None declared.

